# Habitat Stability Modulates Temporal β‐Diversity Patterns of Seagrass‐Associated Amphipods Across a Temperate–Subtropical Transition Zone

**DOI:** 10.1002/ece3.70708

**Published:** 2024-12-12

**Authors:** Sandra Navarro‐Mayoral, Francisco Otero‐Ferrer, Victoria Fernandez‐Gonzalez, Néstor E. Bosch, Yolanda Fernández‐Torquemada, Fiona Tomás, Jorge Terrados, Luis Miguel Ferrero Vicente, Yoana del Pilar‐Ruso, Fernando Espino, Fernando Tuya

**Affiliations:** ^1^ Grupo en Biodiversidad y Conservación, IU‐Ecoaqua Universidad de Las Palmas de Gran Canaria Canary Islands Spain; ^2^ Department of Marine Science and Applied Biology University of Alicante Alicante Spain; ^3^ Instituto Mediterráneo de Estudios Avanzados IMEDEA (CSIC‐UIB) Esporles Spain

**Keywords:** community assembly, ecosystem engineers, environmental stability, epifauna, nestedness, species turnover

## Abstract

Identifying drivers that shape biodiversity across biogeographical regions is important to predict ecosystem responses to environmental changes. While β‐diversity has been widely used to describe biodiversity patterns across space, the dynamic assembly of species over time has been comparatively overlooked. Insights from terrestrial and marine studies on temporal β‐diversity has mostly considered environmental drivers, while the role of biotic mechanisms has been largely ignored. Here, we investigated patterns of temporal variation in β‐diversity of seagrass‐associated amphipods. We conducted a study in three biogeographical regions across a temperate to subtropical latitudinal gradient (approximately 2000 km, 13° of latitude in total). In each region, we randomly selected three *Cymodocea nodosa* meadows, totaling nine meadows sampled seasonally (i.e., four times per year) from 2016 to 2018. We partitioned temporal β‐diversity into its turnover (i.e., species replacement) and nestedness (i.e., differences in species composition caused by species losses) components and addressed the relative influence of both temporal variation in habitat structure (i.e., biotic driver) and environmental conditions on the observed β‐diversity patterns. Our study revealed high temporal β‐diversity of amphipod assemblages across the three biogeographical regions, denoting significant fluctuations in species composition over time. We identified species turnover as the primary driver of temporal β‐diversity, strongly linked to temporal variability in local habitat structure rather than to regional climatic drivers. Subtropical Atlantic meadows with high structural stability over time exhibited the largest turnover rates compared with temperate Mediterranean meadows, under lower structural stability, where nestedness was a more relevant component of temporal β‐diversity. Our results highlight the crucial role of habitat stability in modulating temporal β‐diversity patterns on animals associated with seagrasses, stressing the importance of monitoring variations in habitat structure over time for developing management plans and restoration actions in the context of diversity loss and fragmentation of ecosystems.

## Graphical abstract

This study examines how environmental variation and habitat structure influence temporal patterns of seagrass‐associated fauna, sampling nine meadows at nine different times across regions spanning 2000 km of coastline and two ocean basins. For the first time, we demonstrate the crucial role of temporal changes in habitat structure on β diversity.

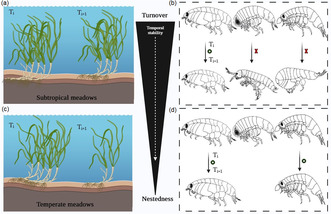

## 1 Introduction

Understanding patterns in variation of biological assemblages and underlying processes has been, for many decades, at the core of ecological research (Brown 1995; Gaston, Chown, and Evans 2008; Gotelli et al. 2009; Bosch et al. 2021). The distribution of biota, with some taxa occurring at certain sites and times but not at others, has significant implications for biodiversity conservation and the maintenance of ecosystem functions (Cardinale et al. 2006; Clare, Culhane, and Robinson 2022). Sites hold varying abundances of different species because of abiotic and biotic filters, which coexist to create a biological community (Brown 1984). As a result, communities across spatial and temporal scales can range from being nearly identical (i.e., when they host the same species) to entirely distinct (i.e., when they harbor completely different species; Baselga and Rodríguez 2019). A key question remains then on how to measure biodiversity to understand the processes that generate such variation in ecological pattern. For example, β‐diversity, often defined as variation in species composition across sites within a geographic area, has been widely used to establish a connection between local (i.e., alpha) and regional (i.e., gamma) diversity patterns (Whittaker 1960; Anderson et al. 2011; Gaggiotti et al. 2018). Despite this spatial approach provides critical information about the structure of communities to inform conservation planning (Socolar et al. 2016), it often overlooks the highly dynamic assembly of species resulting from varying processes that occur over time (Legendre 2019).

In recent years, several studies have adopted a temporal perspective to examine β‐diversity (Baselga, Bonthoux, and Balent 2015; Legendre 2019; Magurran et al. 2019), fueled by the ongoing rapid reorganization of biological assemblages in the Anthropocene (Hillebrand et al. 2018; Blowes et al. 2019; Bosch et al. 2022). This approach evaluates variation in relative abundances and community composition over time and determines whether these differences arise from changes in the identity of species (i.e., replacement or turnover) or from the ordered loss of some species at some points over time (i.e., nestedness) (Baselga 2010). Most insights of temporal trends in β‐diversity, considering its partition into turnover and nestedness components, have been derived from terrestrial ecosystems (Baselga, Bonthoux, and Balent 2015; Uchida and Ushimaru 2015; Legendre and Condit 2019; Crabot et al. 2020; Lindholm et al. 2021; Wu et al. 2022). In marine ecosystems, however, these studies remain scarce and have primarily focused on pelagic organisms, such as bacteria (Hatosy et al. 2013), phytoplankton (Guelzow, Dirks, and Hillebrand 2014), and zooplankton (Lopes et al. 2019). Differently from what usually happens in pelagic ecosystems, the dynamics of benthic ones are primarily shaped by the interplay between abiotic and biotic drivers, particularly for fauna living in close association with the benthos that rely on habitat structural properties. Yet, studies to date on fishes (Magurran et al. 2019; Zeni et al. 2020; Alabia et al. 2021; Camara et al. 2023) has mostly used environmental conditions (e.g., nutrient concentration, temperature, depth) to infer drivers of temporal variability in β‐diversity, therefore ignoring variability in the benthic habitat structure. This reflects the existing gap in our comprehension of biotic drivers influencing temporal β‐diversity in marine ecosystems (i.e., turnover and nestedness components), and questions whether these findings can be generalized to animals with lower mobility and higher dependence on habitat properties.

Benthic marine ecosystems exhibit a high degree of dynamism through both spatial and temporal dimensions (Underwood and Fairweather 1989; Coma et al. 2000). This is particularly evident for diversity patterns of fauna associated with habitats created by “ecosystems engineers” (e.g., kelps, rhodoliths, seagrasses), because associated biotas are intrinsically linked to variation in the structural properties of their habitats (Jacobucci, Tanaka, and Leite 2009; Machado et al. 2019; Navarro‐Mayoral et al. 2020; Pérez‐Peris et al. 2023; Navarro‐Mayoral et al. 2024). In this regard, seagrasses provide a unique environment for studying species assemblages over both space and time, because of their notable variability in abundance and distribution both spatially (e.g., fragmentation across space) and temporally (e.g., seasonal and multiannual dynamics) (Edgar 1990; Boström and Bonsdorff 1997; Guidetti et al. 2002). In general, these marine plants show a marked seasonal variation, with more vigorous meadows in spring and summer, compared to winter and autumn, where they reach minimum vitality (Tuya, Martín, and Luque 2006; Máñez‐Crespo et al. 2020). Moreover, seagrass meadows provide substantial support for epiphytes (Orth, Heck, and van Montfrans 1984; Hall and Bell 1988), which experience temporal fluctuations in abundance and composition, influenced by a range of abiotic (e.g., temperature variations; Borowitzka, Lavery, and Keulen 2006) and biological factors (e.g., intensity of herbivory; Tomas, Turon, and Romero 2005). This makes seagrass meadows one of the marine ecosystems with highest structural and biological heterogeneity (Boström, Jackson, and Simenstad 2006).

Epifauna living on seagrasses typically display large seasonal variation in both abundance and species richness (Moore and Hovel 2010; Leopardas, Uy, and Nakaoka 2014), including mollusks, polychaetes, and crustaceans (e.g., Gambi et al. 1992; Scipione et al. 1996; Nakaoka, Toyohara, and Matsumasa 2001). Amphipods, in particular, are one of the most abundant and diverse animal groups associated with seagrasses (Hyndes and Lavery 2005; Vázquez‐Luis, Sanchez‐Jerez, and Bayle‐Sempere 2009; Michel et al. 2015; Sweatman, Layman, and Fourqurean 2017). These crustaceans are a key group of organisms contributing to energy transfer in marine systems, acting as a link between primary producers and secondary consumers (Myers and Heck Jr 2013), serving as a direct food source for carnivorous decapods and fish (Pinnegar and Polunin 2000). The distribution and diversity of amphipods are influenced by a range of factors, including both habitat structure and environmental processes. For instance, changes in salinity and/or temperature can alter reproduction and survival of amphipods (Welton and Clarke 1980; Sainte‐Marie 1991; Reynolds et al. 2018). However, the structural attributes of the habitat can play an equally, if not more, crucial role than climatic conditions (Fraser et al. 2020; Navarro‐Mayoral et al. 2023). Structural elements of seagrass meadows (e.g., shoot densities) and the availability of secondary substrates (e.g., epiphyte biomass) can profoundly influence the distribution and diversity of amphipods. In other words, these habitat‐related factors interact with climatic conditions over time to shape the community dynamics of amphipod assemblages (Navarro‐Mayoral et al. 2023). Therefore, it is important to consider the temporal component of changes in species composition (i.e., β‐diversity) to improve our understanding of how biotic communities respond in nonstationary environments. Contrary to marine taxa living in more homogenous environments (e.g., plankton) and with higher dispersal abilities (e.g., fishes), amphipods represent an ideal model taxon due to two key aspects of their ecology: (i) their direct development and (ii) low dispersal (Kolding and Fenchel 1981; Sainte‐Marie 1991; Fernandez‐Gonzalez, Navarro‐Mayoral, and Sanchez‐Jerez 2021). Their direct development means they reproduce without a dispersive larval phase, which enhances the stability and local persistence of their populations (Highsmith and Coyle 1991; Fernandez‐Gonzalez, Navarro‐Mayoral, and Sanchez‐Jerez 2021). Additionally, their limited dispersal restricts their spatial movement, heightening their sensitivity to changes within their local habitat (Munguia, Mackie, and Levitan 2007). Consequently, they serve as perfect indicators of the temporal variability of β‐diversity in these ecosystems.

This study aims to investigate patterns and drivers of variation in β‐diversity of seagrass *Cymodocea nodosa*‐associated amphipods over 2 years across a wide biogeographical range, spanning 2000 km across the Atlanto‐Mediterranean region. The distribution of *C. nodosa* encompasses different ecoregions, subjected to varying climatic conditions and landscape configurations (Tuya et al. 2019; Máñez‐Crespo et al. 2020). This provides an ideal case study to partition the influence of both habitat structural attributes and climatic conditions on temporal compositional changes of seagrass‐associated amphipods. In particular, we investigated (1) how β‐diversity varies in amphipod assemblages over time, at 9 times over 2 years at nine meadows from three different marine ecoregions, (2) whether these changes result from the turnover or nestedness components, and (3) which drivers typifying meadow structure and climatic conditions majorly contributed to explain such patterns in temporal β‐diversity. In brief, the goal of this study is to examine temporal changes in β‐diversity of amphipods to identify underlying causes.

## 2 Materials and Methods

### 2.1 Study Areas and Sampling Design

**FIGURE 1:**
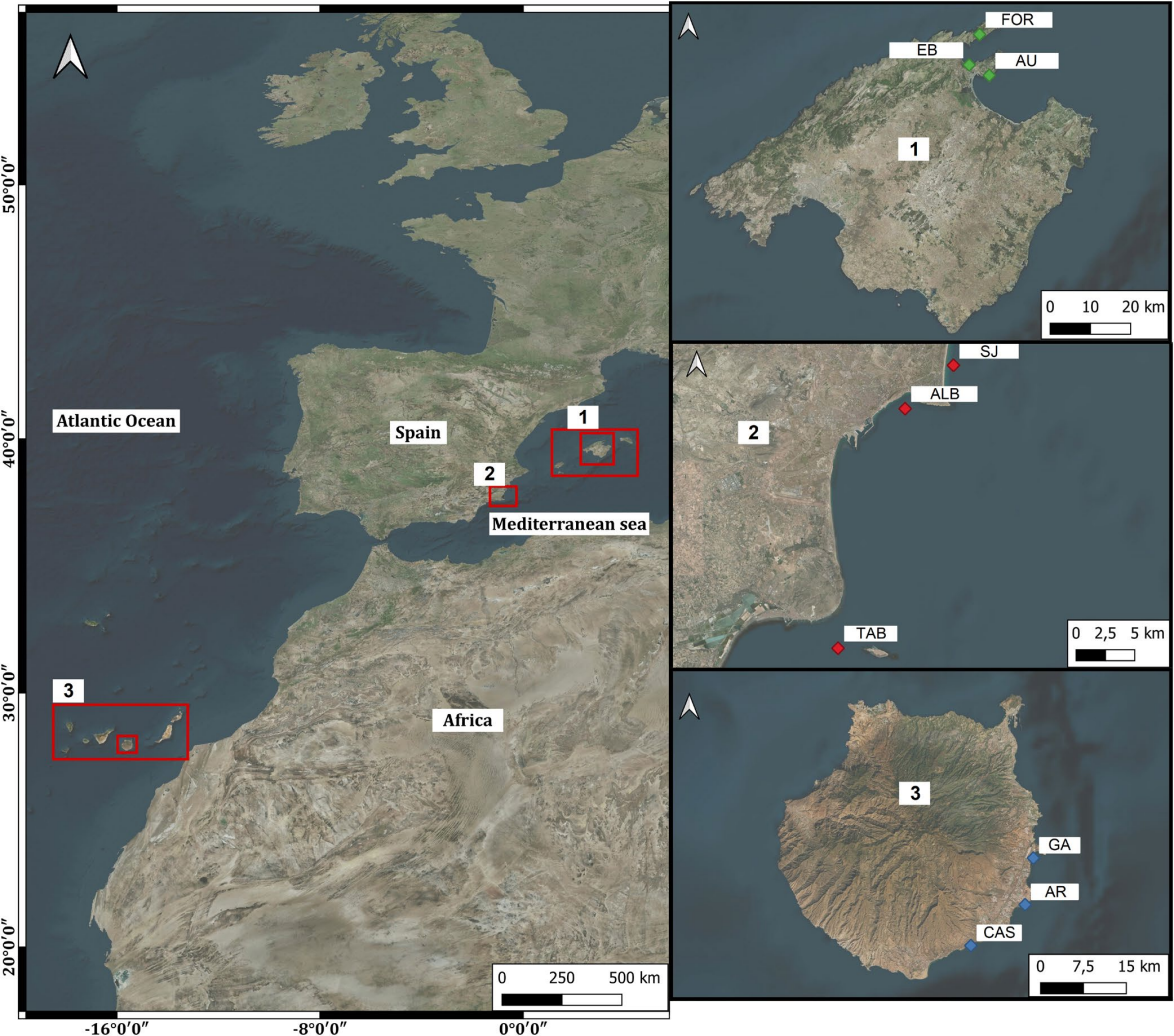
Map of the three biogeographical regions, where amphipod assemblages and seagrass structure were studied. The two regions in the NW Mediterranean are: (1) Mallorca Island (ML) and (2) Alicante (AL); the region in the NE Atlantic is (3) Gran Canaria Island (GC). In each region, three meadows were randomly chosen to capture the variety of meadows within each region.

*Cymodocea nodosa* (Ascherson, 1869) is a dominant seagrass in subtidal zones across the Mediterranean Sea and the adjacent Atlantic Ocean, including southern Portugal, Mauritania, Senegal, the Canary Islands, and Madeira (Tuya et al. 2021). We conducted a study in three biogeographical regions across a temperate to subtropical latitudinal gradient (~2000 km, 13° of latitude; Figure 1). This distribution covered the eastern Iberian Peninsula (Alicante [AL], 38° N), the Balearic Sea (Mallorca Island [ML], 40° N), both regions in the Western Mediterranean, and Macaronesia (Gran Canaria Island [GC], 28° N) in the northeastern Atlantic Ocean (Figure 1). The study regions represent the full range of climate types within the geographic distribution of *C. nodosa* , according to Köppen‐Geiger classification (Kottek et al. 2006). Specifically, Alicante features a hot semiarid climate (BSh) characterized by mild winters and hot summer with minimal rainfall; Mallorca has a warm temperature climate with dry summers (Csa); and Gran Canaria exhibits a hot desert climate (BWh) with mild temperatures year‐round.

In each region, we randomly chose three nearshore *C. nodosa* meadows, each separated by at least 4 km, to capture variations in meadow structure and environmental conditions within each biogeographical region. In Mallorca (ML), the meadows chosen were Formentor (FOR), Es Barcares (EB), and Aucanada (AU). In Alicante (AL), we selected San Juan (SJ), Albufereta (ALB), and Tabarca (TAB), while in Gran Canaria (GC), the meadows were Gando (GA), Arinaga (AR), and Castillo (CAS). At each meadow, seagrass structure and amphipod assemblages were sampled seasonally (i.e., 4 times a year) throughout 2016 to 2018, for a total of nine temporal collections at each of the nine meadows.

### 2.2 Collection of Amphipod Assemblages

At each time, we collected five random samples of vegetation and associated epifauna, separated by ~5 m from each other (Edgar and Robertson 1992). We used a fine mesh bag (250 μm) affixed to a quadrat (25 × 25 cm) placed over the seagrass canopy (0.0625 m^2^ of total area), which was then cut at the sediment surface level. We used this specific mesh size to effectively capture all amphipods, given their typical size range of 500 μm to 1 mm (Hughes and Ahyong 2016). Sample bags were transported to the laboratory, where amphipods were separated using a 500‐μm mesh, and then identified and counted under a stereomicroscope (Ruffo 1998). For each sample, we determined species abundances (expressed as number of individuals per m^2^).

### 2.3 Habitat Structure and Climatic Context

For each sampling time and meadow, seagrass cover was in situ estimated by deploying on the bottom a 25‐m‐long fiberglass transect, and subsequent annotation of the total distance covered by the seagrass; final values were then expressed in percent cover. Plant biomass was measured by taking *n* = 10 cores (20 cm inner diameter, 50 cm depth) haphazardly located within each meadow. In the laboratory, sediment was removed from the cores, and aboveground biomass was separated and dried at 60°C for 48 h. Seagrass leaf biomass data were standardized to the core area and expressed as g DW cm^−2^. Seagrass density was estimated by counting the number of shoots in a 20 × 20 cm^2^ quadrant (*n* = 10) haphazardly allocated at each meadow and time. The density of shoots was expressed per m^2^. In addition, 20 shoots were randomly collected by hand at each meadow and sampling time. In the laboratory, we quantified the number of leaves per shoot, as well as the length and width (mm) of all leaves. Macroscopic epiphytes were removed using a razor blade and epiphytes and leaves were subsequently oven‐dried to estimate epiphytic load (i.e., dry weight, DW, of epiphytes per DW of leaf biomass). Total leaf area (seagrass surface area; SSA) was obtained as the sum of all the individual leaf areas of all leaves per shoot (cm^2^ shoot^−1^), and the leaf area index (LAI) was estimated by multiplying the total leaf area per shoot by the mean shoot density per meadow and time. Epiphytic loads were expressed as g DW of epiphytes per g DW of leaf. These data were already presented in Máñez‐Crespo et al. (2020).

To describe spatial and temporal variability in ocean climate, monthly data of sea surface temperature (SST) and photosynthetically active radiation (PAR) intensity were obtained through the entire study period (2016–2018), as in Máñez‐Crespo et al. (2020), from the moderate resolution imaging spectroradiometer facility (MODIS‐Aqua), using the Nasa Giovanni system (https://giovanni.gsfc.nasa.gov/giovanni/). The spatial resolution of the SST and PAR gridded layers, that encompassed all the nine surveyed meadows, was a 4‐km/pixel (i.e., 16 km^2^). For each sampling period, we calculated the mean SST and PAR at the meadow scale for each biogeographical region.

### 2.4 Statistical Analyses

#### 2.4.1 Temporal β‐diversity of Amphipod Assemblages

Differences in the composition and structure of amphipod assemblages were assessed for each meadow over the nine times using the “betapart” R package (Baselga and Orme 2012). For species composition, we used the “beta.multi” function (Baselga 2010) with the “Sørensen” family of dissimilarity (*β*_SOR_; Equation (1)). This approach partitioned temporal β‐diversity into its turnover (*β*_SIM_: turnover component of Sørensen dissimilarity; Equation (2)) and nestedness (*β*_SNE_: nestedness component of Sørensen dissimilarity; Equation (3)) components. When considering abundances, the “beta.multi.abun” function was used, with the Bray–Curtis dissimilarity family specified (Equation (4)). Hence, total dissimilarity for each meadow through time was studied by considering abundance data in addition to composition. The equations to calculate indices of temporal β‐diversity for multiples times considering the composition and structure data were the following:

Sørensen dissimilarity for multiple times:

(1) $$\beta_{\mathrm{SOR}}=\frac{\left[\sum_{i<j}\min\left(b_{\mathrm{ij}},b_{\mathrm{ji}}\right)\right]+\left[\sum_{i<j}\max\left(b_{\mathrm{ij}},b_{\mathrm{ji}}\right)\right]\;}{2\left[\sum_{i}S_{i}-S_{t}\right]+\left[\sum_{i<j}\min\left(b_{\mathrm{ij}},b_{\mathrm{ji}}\right)\right]+\left[\sum_{i<j}\max\left(b_{\mathrm{ij}},b_{\mathrm{ji}}\right)\right]}$$

where *S*_i_ is the total number of species in time *i*, *S*_T_ is the total number of species at all times, and *b*_*ij*_, *b*_*ji*_ are the number of species exclusive to times *i* and *j*, respectively, when compared by pairs.

Dissimilarity component for species turnover for multiple times:

(2) $$\beta_{\mathrm{SIM}}=\frac{\left[\sum_{i<j}\min\left(b_{\mathrm{ij}},b_{\mathrm{ji}}\right)\right]}{\left[\sum_{i}S_{i}-S_{T}\right]+\left[\sum_{i<j}\min\left(b_{\mathrm{ij}},b_{\mathrm{ji}}\right)\right]}$$

Dissimilarity component for species nestedness for multiple times:

(3) $${Β}_{\mathrm{NES}}=\beta_{\mathrm{SOR}}—\beta_{\mathrm{SIM}}$$

Bray–Curtis dissimilarity for multiple times:

(4) $$\mathrm{BC}=\frac{\sum_{k=1}^{s}\min\left(x_{\mathrm{ik}},\;\;x_{\mathrm{jk}}\right)}{\sum_{k=1}^{S}\left(x_{\mathrm{ik}}+x_{\mathrm{jk}}\right)}$$

where *S* is the total number of species, *x*_*ik*_ represents the abundance of species *k* at time *i*, and *x*_*jk*_ represents the abundance of species *k* at time *j*. The numerator sums the minimum abundances of each species between times *i* and *j*, while the denominator sums the total abundances of the species across both times.

#### 2.4.2 Drivers of Temporal β‐diversity

Univariate generalized linear models (GLMs) were implemented to explore the relative contribution of predictor variables, typifying temporal variation (via coefficients of variation) in the habitat structure and environment of each seagrass meadow, on variation in the components of temporal β‐diversity of amphipod assemblages (i.e., Sørensen, turnover, nestedness, and Bray–Curtis index). Prior to implementation of the models, correlations (Spearman's coefficients) among each pair of predictor variables were tested and visualized using the “corrplot” R package (Wei et al. 2017). To limit the inclusion of overly correlated predictors in the models, we chose those with a larger biological significance among those predictors that were correlated (Spearman's correlation coefficient with *r* ^2^ > 0.6; see Appendix S1 in Supporting Information) (Bolker 2008). This analysis led to the selection of four predictors: seagrass leaf biomass, seagrass cover, seagrass shoot density, and epiphytic loads, as descriptors of habitat (seagrass meadow) structure, and mean seasonal SST within a meadow as a descriptor of ocean climate. Variance inflation factors (VIFs) among predictors were calculated using the “car” R package (Fox et al. 2012): the obtained values were always < 5, indicating that multicollinearity was not a concern for our analyses (Quinn and Keough 2002).

Model selection was performed to identify those predictors, or combinations, that better explain variation in the components of temporal β‐diversity of amphipod assemblages. First, we used the “MASS” R package (Ripley et al. 2013) to perform a backward stepwise approach by iteratively removing from the full model the predictor variable with the lowest contribution, until obtaining the most parsimonious model according to the Akaike's information criterion corrected for small sample sizes (AICc). Then, we used the “MuMIn” R package (Barton and Barton 2015), with the aims of (i) validating the previous model (stepwise) selection by constructing a full set of candidate models (i.e., models containing all combinations of 1, 2, 3, 4, or 5 predictors), ranking models according to the AICc; and (ii) estimating the relative importance of each predictor variable. For all fitted models, diagnosis plots of residuals and Q–Q plots were used to visually inspect their appropriateness, while assumptions of homogeneous variance were checked using the Breusch–Pagan heteroskedasticity test.

## 3 Results

### 3.1 Temporal β‐diversity of Amphipods

**FIGURE 2:**
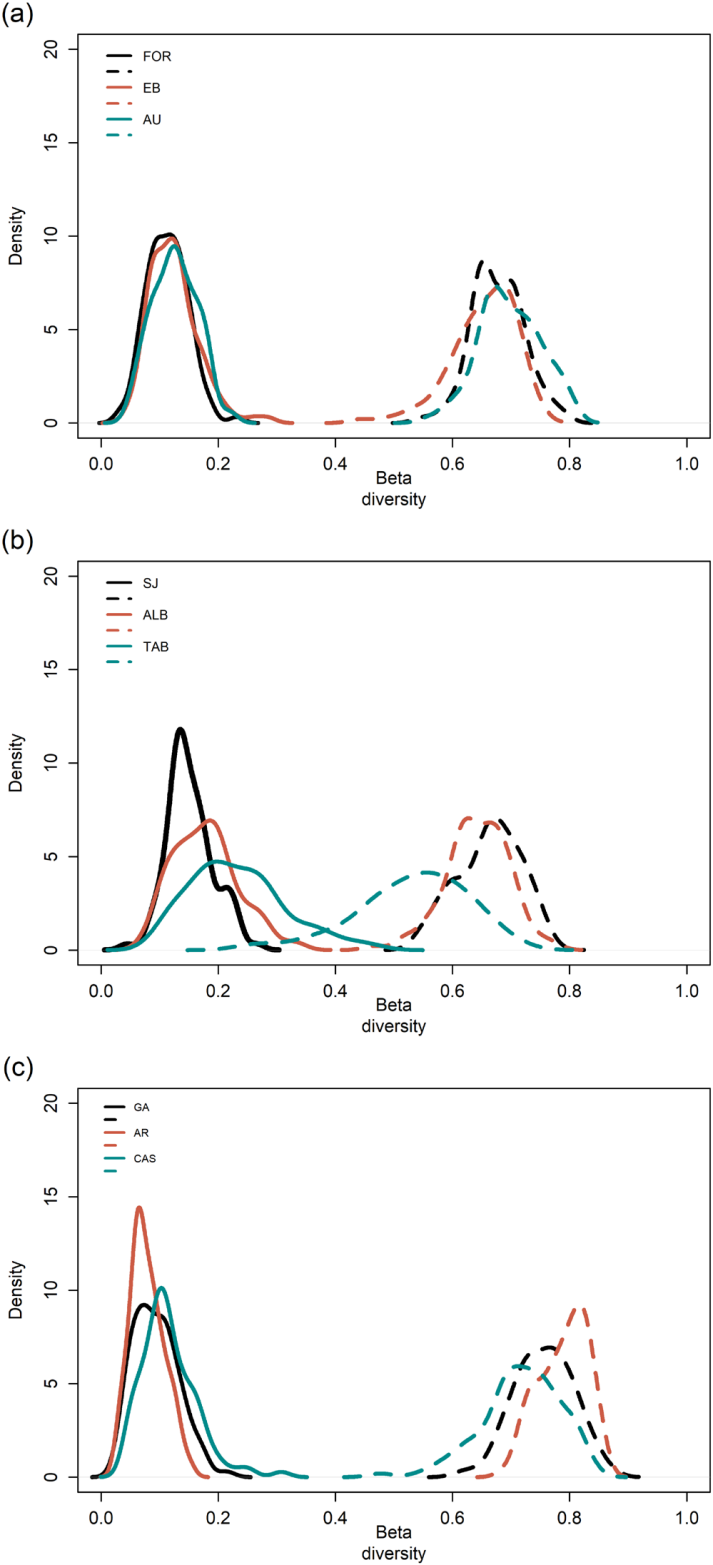
Density plots of temporal components of β‐diversity of amphipod assemblages for each meadow from each of the three biogeographical regions: ML (a), AL (b), and GC (c). Solid and dashed lines denote nestedness and turnover components, respectively.

In total, 6794 amphipods were counted, including 81 taxa (73 identified at the species level and 8 at the genus level; see Appendix S2). We found a high dissimilarity in amphipod composition through time across all three regions, with the Sørensen index displaying high values irrespective of the seagrass meadow (i.e., *β*_SOR_; Table 1). Turnover (*β*_SIM_) was the main contributor to β‐diversity across all meadows, while the nestedness component contributed considerably less to overall compositional differences, even being negligible in some meadows (e.g., GC, < 0.1; Figure 2; Table 1). This implies that, in most meadows, the composition of species significantly varied over time, with replacement (turnover) of species as the primary driver of β‐diversity. GC showed the highest turnover and the lowest nestedness, with a *β*_SIM_ of 0.73 ± 0.03 (mean ± SD) and a *β*_SNE_ of 0.08 ± 0.01. In AL, meadows showed the lowest turnover (*β*_SIM_ of 0.56 ± 0.02) and the highest nestedness (*β*_SNE_ of 0.22 ± 0.06). ML displayed intermediate values for turnover and nestedness (*β*_SIM_ = 0.63 ± 0.02 and *β*_SNE_ = 0.14 ± 0.01, respectively).

**TABLE 1:** Results of components of temporal β‐diversity of seagrass‐associated amphipod assemblages, for each seagrass meadow at each region, including turnover (replacement), and nestedness–resultant components, Sorensen (i.e., the sum of both components), for species composition, and the Bray–Curtis index for species abundances.

Region	Meadow	Sorensen	Turnover	Nestedness	Bray–Curtis
ML	FOR	0.71	0.59	0.12	0.86
ML	EB	0.82	0.68	0.14	0.93
ML	AU	0.80	0.63	0.16	0.92
AL	SJ	0.74	0.60	0.14	0.91
AL	ALB	0.74	0.59	0.15	0.85
AL	TAB	0.87	0.50	0.37	0.89
GC	GA	0.84	0.77	0.07	0.86
GC	AR	0.82	0.73	0.09	0.85
GC	CAS	0.79	0.71	0.08	0.78

### 3.2 Drivers of Temporal β‐diversity

**FIGURE 3:**
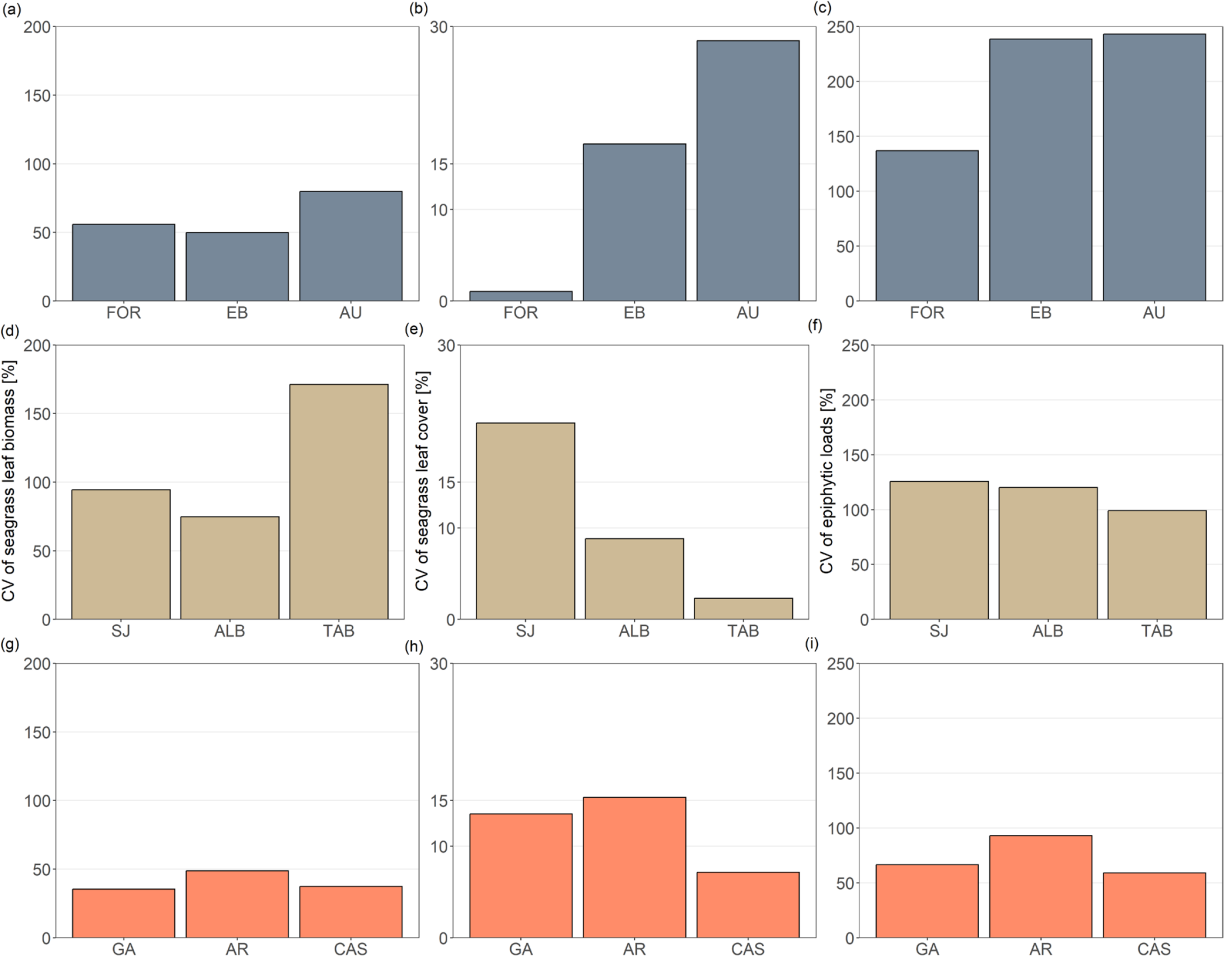
Coefficient of variation (CV) of predictor variables typifying seagrass habitat structure that contributed to explain variation in temporal components of beta biodiversity of amphipod assemblages for ML (a–c), AL (d–f), and GC (g–i).

The overall β‐diversity in amphipod composition (i.e., Sørensen index; Table 2) was primarily determined by seagrass cover, accounting for ~25% of the variance (Table 3). Seagrass leaf biomass was the predictor majorly determining variation in turnover and nestedness in amphipod composition, accounting for 64% and 85% of the variance, respectively (Table 3). However, in the case of turnover, seagrass cover also contributed to explain additional variation, according to the multimodel averaging (Table 3). Overall, a distinct pattern emerged among regions; meadows with a lower coefficient of variation for both seagrass leaf biomass and cover exhibited higher turnover values (Table 1), as in GC (Table 1; Figure 3g,h). None of the environmental (climatic) predictor variables (i.e., SST and PAR) were significant in explaining variability in β‐diversity components or total assemblage abundance.

**TABLE 2:** Predictor variables determining patterns of temporal variation in β‐diversity for the composition (turnover and nestedness) and structure (composition and abundances, Bray–Curtis dissimilarities) of seagrass‐associated amphipods, according to results of model selection from stepwise model selection.

	Estimate	*SE*	*z*	*p*
*Sørensen*				
Intercept	0.82	0.02	38.50	**2.08 e** ^**−09**^
Seagrass cover	−0.003	0.001	−1.93	**0.03**
*Turnover*				
Intercept	0.75	0.03	24.70	**4.54 e** ^**−08**^
Seagrass leaf biomass	−0.001	0.0004	−3.89	**0.0005**
Seagrass cover	0.0002	0.003	0.07	**0.04**
*Nestedness*				
Intercept	0.004	0.02	0.19	0.85
Seagrass leaf biomass	0.002	0.0003	6.74	**0.0001**
*Bray–Curtis*				
Intercept	0.81	0.02	39.06	**1.88 e** ^**−09**^
Epiphytic load	0.0004	0.0001	3.29	**0.008**

*Note:* Significant differences (p < 0.05) are highlighted in bold.

**TABLE 3:** Model selection of predictor variables explaining patterns of temporal variation in β‐diversity for the composition (turnover and nestedness) and assemblage structure (composition and abundances, Bray–Curtis dissimilarities) of amphipod assemblages. Models are ranked according to the AICc; adjusted *R* ^2^, associated *p* values. Results of the Breusch–Pagan heteroskedasticity tests are included.

Variable	Model predictors	df	LogLik	AICc	Delta AICc	Weight (wi)	Adjusted *R* ^2^	*F*	*p*	Breusch–Pagan test
Sorensen	Seagrass cover	3	18.51	−26.2	0.00	0.498	0.25	3.73	0.03	BP = 1.43, df = 1, *p* = 0.23
	Seagrass leaf biomass	3	17.44	−24.1	2.15	0.170	0.05	1.45	0.27	
Turnover	Seagrass leaf biomass	3	16.46	−22.1	0	0.50	0.64	15.16	0.005	BP = 0.30, df = 1, *p* = 0.58
	Seagrass leaf biomass, seagrass cover	4	19.91	−21.8	0.30	0.429	0.80	17.45	0.003	
Nestedness	Seagrass leaf biomass	3	19.20	−27.6	0	0.861	0.85	45.52	0.0002	BP = 1.45, df = 1, *p* = 0.23
Bray–Curtis	Epiphytic load	3	20.81	−30.8	0	0.64	0.55	10.87	0.001	BP = 2.47, df = 1, *p* = 0.11

When considering abundances, all meadows from each region exhibited values of the Bray–Curtis index close to 1 (Table 1), which reflects a high variability in species' abundances over time. The primary predictor accounting for observed variation was the epiphytic loads, which explained ~55% of the total variance (Table 3). In particular, we found greater fluctuations in assemblage structure over time in those regions with larger variation in epiphytic loads (i.e., ML and AL; Table 1), in contrast to GC, which exhibited the smallest differences in assemblage structure and the lowest coefficient of variation in our study (Table 1; Figure 3i).

## 4 Discussion

**FIGURE 4:**
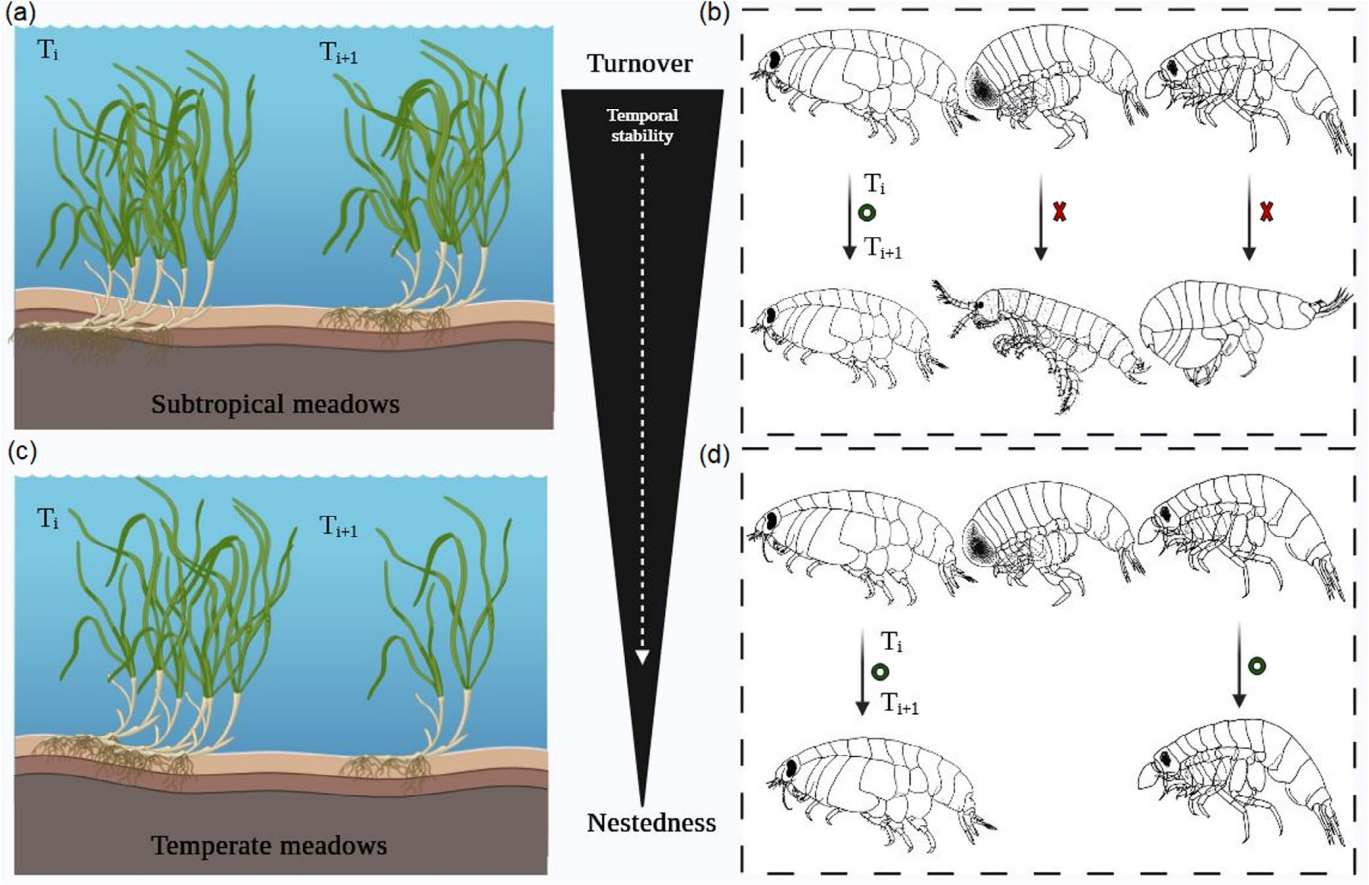
Latitudinal trends in the temporal β‐diversity of amphipods associated with *Cymodocea nodosa* are strongly linked to the stability of seagrass structural properties over time. Species turnover was the main mechanism generating dissimilarity across meadows, exhibiting higher values in subtropical meadows (a, b) compared to temperate ones (c, d), where the nestedness component was important. Red crosses denote temporary turnover and green circles denote temporary nestedness.

We found that amphipod assemblages displayed large temporal β‐diversity in each meadow across the three biogeographical regions. Species turnover was the main process contributing to temporal β‐diversity, which exhibited higher values in subtropical relative to more temperate meadows. Importantly, this disparity in temporal β‐diversity of amphipod assemblages was more linked to local structural properties of meadows than climatic regional drivers (Figure 4). Amphipod turnover typically follows seasonal variation not only in environmental conditions (e.g., temperature, photoperiod), but also in food resources (e.g., macrophyte quality and quantity, in the case of herbivorous amphipods) and energetic requirements directly connected with the habitat structure (Neuparth, Costa, and Costa 2002; Maranhão and Marques 2003). In this sense, it is known that spatial variation in environmental conditions and ecological resources filter species in local communities (Bueno et al. 2016), leading to high rates of species turnover across space. In our case, we assume that, in seagrass meadows, where fluctuations in growth and expansion (i.e., vitality) in the seagrass occur throughout seasons and years (Máñez‐Crespo et al. 2020), similar processes would be occurring to generate temporal patterns in β‐diversity of amphipod assemblages. Thus, temporal variation in structural properties of the habitat affects the diversity of microhabitats and the availability of resources (e.g., epiphytes, detritus, and organic material), filtering amphipod species to be present at different times (Bologna and Heck Jr 1999). For example, during periods of high epiphyte productivity, herbivorous species can proliferate (Michel et al. 2015), whereas in times of greater detritus accumulation, detritivores species can become dominant (Zimmerman, Gibson, and Harrington 1979). Additionally, our study recorded a total of 81 taxa, which is higher than the richness found in other seagrass meadows: 37 taxa (Zakhama‐Sraieb et al. 2006), 76 taxa (Sturaro et al. 2014), and 38 taxa (Navarro‐Mayoral et al. 2023). This increased species richness may be due to the longer duration of our study, as species composition can change with the seasons. By extending the temporal scale, we were able to capture this variability more effectively. Comparing our findings on the temporal variability in β‐diversity of seagrass‐associated amphipods with other studies focusing on this animal group is difficult; only Cereghetti and Altermatt (2023) have explored this with freshwater amphipod assemblages associated with small tributary streams, employing a different statistical framework. Contrary to our results, they did not identify turnover as the main driver of temporal β‐diversity patterns in amphipods. Rather, they found a temporally consistent coexistence of species, with some fluctuations in certain taxa that were mainly due to different uses and intensity of agricultural land uses surrounding streams. Nonetheless, our temporal pattern in β‐diversity across all meadows (i.e., turnover > nestedness) appears to be ubiquitous in nature (i.e., freshwater, marine, and terrestrial realms). For example, according to Soininen, Heino, and Wang (2018), turnover is typically more than five times larger than nestedness. Still, our findings diverge from the usual drivers of these patterns, as higher turnover rates in marine environments are typically associated with increased environmental variability, rather than the reverse pattern (this study). In this regard, studies with marine groups such as microbes (Hatosy et al. 2013) or zooplankton (Lopes et al. 2019) also found species turnover as the primary driver of temporal β‐diversity, which was therein attributed to temporal fluctuations in nutrient concentrations and climatic conditions. In other environments, such as tropical mountain habitats, temporal β‐diversity of ant communities was also driven by replacement of certain species by others over time. However, higher turnover rates were again linked to fluctuations in environmental factors (i.e., greater instability), such as temperature, humidity, and resource availability (Nunes et al. 2020).

We found that the contribution of species turnover to dissimilarity over time was somehow not consistent across regions. These inter‐regional differences were mainly explained by temporal variation in the structural attributes of the meadows, specifically seagrass leaf biomass and cover. Of these, seagrass leaf biomass was the most influential variable, explaining ~64% in variation of species turnover. In this sense, the highest turnover values were found in the subtropical region (contributing on averaged ~73%), coinciding with the lowest coefficient of variation of seagrass leaf biomass. In contrast, temperate regions presented lower turnover on average (~66% in ML and ~57% in AL), coinciding with greater temporal variability in the structure of these meadows. On the other hand, the contribution of nestedness was low across all meadows, except for TAB meadow (AL region), where the value was high, coinciding with the highest temporal variability of seagrass leaf biomass. Interestingly, these results indicate that more stable meadows over time (i.e., with lower coefficient of variation in their structural attributes) drove higher turnover values. These findings stress the importance of temporal variability in local habitat structure in mediating β‐diversity patterns of animal assemblages, a key element that has been overlooked, to the best of our knowledge, in terrestrial (Cook et al. 2018; García‐Llamas et al. 2019; Zeni et al. 2020; Wu et al. 2022) and marine (Hatosy et al. 2013; Guelzow, Dirks, and Hillebrand 2014; Lamy et al. 2015; Alabia et al. 2021) studies conducted to date. Previous works have demonstrated that the contribution of species turnover to spatial β‐diversity is greatest in those regions under stable climates (Baselga, Gómez‐Rodríguez, and Lobo 2012; Dobrovolski et al. 2012). For example, amphibian assemblages in “unstable areas” were dominated by “nested” species losses that lead to high nestedness–resultant dissimilarity, while species replacements that lead to high spatial turnover were the predominant process in “stable areas” over evolutionary timescales (Baselga, Gómez‐Rodríguez, and Lobo 2012). However, in contemporary timescales, decreased compositional stability (i.e., high turnover rates) has been associated with an increase in environmental instability (La Sorte, Tingley, and Hurlbert 2014; Hillebrand, Soininen, and Snoeijs 2010; He et al. 2024). Our findings diverge from the patterns described in the literature (Hatosy et al. 2013; Lopes et al. 2019, Nunes et al. 2020), likely because we focused on the role of biotic mechanisms in temporal β‐diversity rather than on abiotic factors. The increased nestedness rates observed in temperate meadows suggest that temporal variation in the structural properties of habitats can act as a filter, impacting the persistence of certain species and potentially leading to the loss of rare species (Davies, Margules, and Lawrence 2004). Rare species, characterized by their low abundance and limited regional occupancy, can be particularly vulnerable to these environmental changes (Foden et al. 2019). This vulnerability may lead to the persistence of dominant competitive species in unstable areas, with rare species potentially being replaced by opportunistic counterparts. In contrast, in more stable environments like the subtropical meadows, both dominant and rare species experience changes, contributing to a heightened turnover rate (Setubal and Bozelli 2021).

We also found an inter‐regional pattern in temporal β‐diversity when considering abundances, which was driven by variation in epiphytic loads. In this case, the temperate meadows showed greater dissimilarity over time, coinciding with the greater temporal variability of epiphytes. This result was expected, since the availability of food resources (e.g., epiphytes) stands out as one of the most influential factors that shape amphipod abundances (Cook et al. 2018; Michel et al. 2015). It is known that epiphytes usually respond to environmental changes more quickly than the seagrasses themselves (Borum 1985; Frankovich et al. 2009). Thus, climate stability in the subtropical region leads to lower temporal variation of epiphytes on *C. nodosa* leaves, providing a stable resource that positively influences various aspects, such as space availability (Osman 1977; Leite, Tanaka, and Gebara 2007), food sources (Edgar 1990; Buzá‐Jacobucci and Pereira‐Leite 2014), refuge provision (Leber 1985; Tuya, Larsen, and Platt 2011), and predator–prey dynamics (Orth, Heck, and van Montfrans 1984; Alexander et al. 2012). In contrast, the dynamic nature of temperate meadows, subjected to higher fluctuations of temperatures and light regimes, produces greater variations in the epiphytic load (Balata et al. 2007), which increased demands of certain species (e.g., herbivorous amphipods) reliant on epiphytes for survival.

## 5 Conclusions

Our study is, to the best of our knowledge, the first to directly examine the effect of temporal changes in habitat structure on temporal β‐diversity of associated fauna. Results highlight that temporal β‐diversity of amphipod assemblages is sensitive to variability in the structure of the habitat provided by the seagrass *Cymodocea nodosa*. We evidenced that species turnover emerges as the primary process driving temporal β‐diversity, with a higher prevalence in subtropical meadows under large structural habitat stability. Given that, to date, most studies have linked the replacement of some species by other to environmental instability, more studies are necessary to understand the crucial role of habitat stability in sustaining both long‐term resident species (i.e., specialized species) and transient species, mainly in a context of loss of diversity and fragmentation of ecosystems.

## Author Contributions

**Sandra Navarro‐Mayoral:** conceptualization (equal), data curation (lead), formal analysis (lead), investigation (equal), methodology (equal), writing – original draft (equal). **Francisco Otero‐Ferrer:** investigation (equal), supervision (lead), validation (equal), writing – review and editing (equal). **Victoria Fernandez‐Gonzalez:** supervision (equal), writing – review and editing (equal). **Néstor E. Bosch:** conceptualization (equal), validation (equal), writing – review and editing (equal). **Yolanda Fernández‐Torquemada:** investigation (equal), methodology (equal), writing – review and editing (equal). **Fiona Tomás:** investigation (equal), methodology (equal), writing – review and editing (equal). **Jorge Terrados:** investigation (equal), methodology (equal), writing – review and editing (equal). **Luis Miguel Ferrero Vicente:** investigation (supporting), writing – review and editing (supporting). **Yoana del Pilar‐Ruso:** investigation (supporting), writing – review and editing (equal). **Fernando Espino:** investigation (equal), writing – review and editing (equal). **Fernando Tuya:** conceptualization (lead), funding acquisition (lead), investigation (lead), methodology (equal), project administration (equal), supervision (lead), validation (equal), visualization (equal), writing – review and editing (equal).

## Conflicts of Interest

The authors declare no conflicts of interest.

## Supporting information


Appendix S1.

## Data Availability

Data available from the figshare repertory: https://doi.org/10.6084/m9.figshare.26303875.v1.
