# Supplementary material for: Habitat Stability Modulates Temporal β‐Diversity Patterns of Seagrass‐Associated Amphipods Across a Temperate–Subtropical Transition Zone
Source: Ecol Evol. 2024 Dec 12;14(12):e70708. doi: 10.1002/ece3.70708 (PMC11635179; doi:10.1002/ece3.70708)
Supplement: Supplementary file 1 — Appendix S1. [file ECE3-14-e70708-s001.docx]

**Supporting Information**


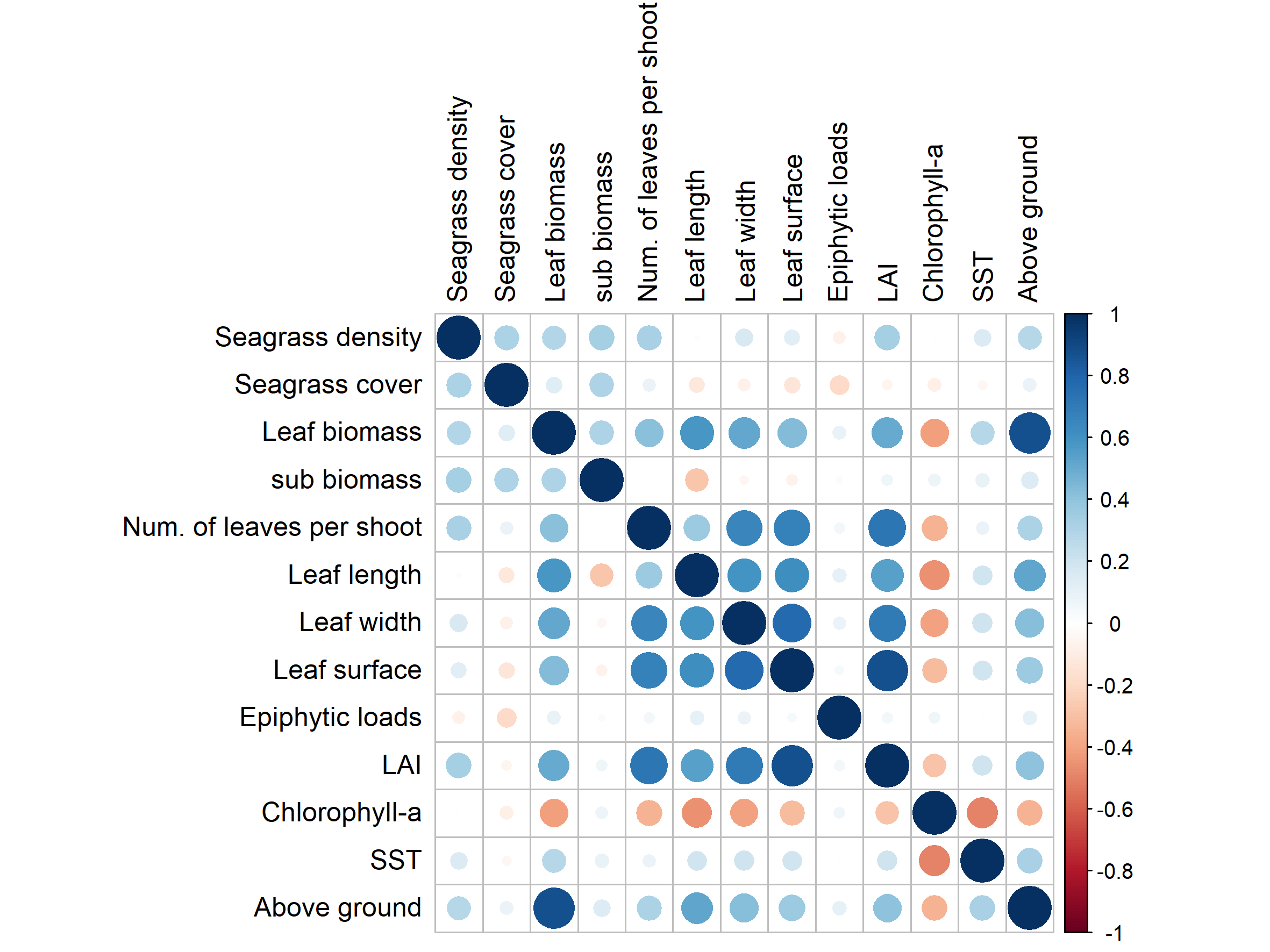


**Appendix S1.** Spearman product-moment correlation matrix between each pair of predictor variables. The bigger and darker the colour, the stronger the correlation. Seagrass leaf biomass (Leaf biomass), Number of leaves per shoot (Num. of leaves per shoot), mean seasonal Sea Surface Temperature (SST) and Leaf Area Index (LAI).

**Appendix S2.** List of amphipod species observed in the *C. nodosa* meadows over the two-year study period.

| **Family** | **Species** |
| --- | --- |
| Ampeliscidae | *Ampelisca ledoyeri* |
| Ampeliscidae | *Ampelisca spinipes* |
| Ampeliscidae | *Ampelisca sarsi* |
| Amphilochidae | *Amphilochus brunneus* |
| Amphilochidae | *Amphilocus manudens* |
| Ampithoidae | *Ampithoe helleri* |
| Ampithoidae | *Ampithoe ramondi* |
| Maeridae | *Animoceradocus semiserratus* |
| Aoridae | *Aora spinicornis* |
| Calliopiidae | *Apherusa alacris* |
| Calliopiidae | *Apherusa bispinosa* |
| Calliopiidae | *Apherusa chiereghinii* |
| Calliopiidae | *Apherusa mediterranea* |
| Calliopiidae | *Apherusa ruffoi* |
| Calliopiidae | *Apherusa vexatrix* |
| Calliopiidae | *Apherusa sp* |
| Argissidae | *Argissa hamatipes* |
| Atylidae | *Nototropis guttatus* |
| Atylidae | *Nototropis massiliensis* |
| Atylidae | *Nototropis swammerdamei* |
| Atylidae | *Nototropis vedlomensis* |
| Bathyporeiidae | *Bathyporeia guilliamsoniana* |
| Caprellidae | *Caprella acanthifera* |
| Caprellidae | *Caprella sp* |
| Maeridae | *Animoceradocus semiserratus* |
| Corophiidae | *Monocorophium insidiosum* |
| Dexaminidae | *Dexamine spinosa* |
| Maeridae | *Elasmopus pocillimanus* |
| Maeridae | *Elasmopus sp* |
| Ischyroceridae | *Ericthonius difformis* |
| Ischyroceridae | *Ericthonius punctatus* |
| Pontogeneiidae | *Eusiroides dellavallei* |
| Nuuanuidae | *Gammarella fucicola* |
| Photidae | *Gammaropsis dentata* |
| Photidae | *Gammaropsis maculata* |
| Photidae | *Gammaropsis palmata* |
| Phoxocephalidae | *Harpinia agna* |
| Phoxocephalidae | *Harpinia ala* |
| Hyalidae | *Lelehua sp* |
| Iphimediidae | *Iphimedia carinata* |
| Iphimediidae | *Iphimedia gibbula* |
| Iphimediidae | *Iphimedia minuta* |
| Iphimediidae | *Iphimedia obesa* |
| Iphimediidae | *Iphimedia vicina* |
| Ischyroceridae | *Jassa marmorata* |
| Ischyroceridae | *Jassa slatteryi* |
| Aoridae | *Bemlos virgus* |
| Aoridae | *Lembos sp* |
| Corophiidae | *Leptocheirus bispinosus* |
| Corophiidae | *Leptocheirus mariae* |
| Corophiidae | *Leptocheirus pectinatus* |
| Corophiidae | *Leptocheirus sp* |
| Leucothoidae | *Leucothoe oboa* |
| Leucothoidae | *Leucothoe richiardii* |
| Photidae | *Megamphopus longicornis* |
| Megaluropidae | *Megaluropus massiliensis* |
| Phoxocephalidae | *Metaphoxus simplex* |
| Aoridae | *Microdeutopus anomalus* |
| Aoridae | *Microdeutopus bifidus* |
| Aoridae | *Microdeutopus chelifer* |
| Aoridae | *Microdeutopus obtusatus* |
| Aoridae | *Microdeutopus similis* |
| Aoridae | *Microdeutopus stationis* |
| Aoridae | *Microprotopus longimanus* |
| Aoridae | *Microdeutopus sp* |
| Microprotopidae | *Microprotopus maculatus* |
| Microprotopidae | *Microprotopus sp* |
| Cyproideidae | *Peltocoxa marioni* |
| Cyproideidae | *Peltocoxa mediterranea* |
| Phliantidae | *Pereionotus testudo* |
| Oedicerotidae | *Perioculodes longimanus angustipes* |
| Oedicerotidae | *Perioculodes longimanus longimanus* |
| Photidae | *Photis lamellifera* |
| Photidae | *Photis longipes* |
| Caprellidae | *Phtisica marina* |
| Caprellidae | *Pseudoprotella phasma* |
| Ischyroceridae | *Centraloecetes bulborostrum* |
| Lysianassidae | *Socarnes filicornis* |
| Stenothoidae | *Stenothoe monoculoides* |
| Urothoidae | *Urothoe intermedia* |
|  |  |
